# Improvement in Pulmonary Function with Short-term Rehabilitation Treatment in Spinal Cord Injury Patients

**DOI:** 10.1038/s41598-019-52526-6

**Published:** 2019-11-19

**Authors:** Ji Cheol Shin, Eun Young Han, Kye Hee Cho, Sang Hee Im

**Affiliations:** 1 Department and Research Institute of Rehabilitation Medicine, Severance Hospital, Yonsei University College of Medicine, Seoul, Republic of Korea; 2 Department of Rehabilitation Medicine, Jeju National University Hospital, Jeju National University School of Medicine, Jeju, Republic of Korea; 3 Department of Rehabilitation Medicine, CHA Gumi Medical Center, CHA University, Gumi, Gyeongsangbukdo Republic of Korea

**Keywords:** Patient education, Spinal cord diseases

## Abstract

Cervical and upper thoracic spinal cord injury causes impairments in respiratory muscle performance, leading to variable degrees of pulmonary dysfunction and rendering deep breathing difficult for affected individuals. In this retrospective study, we investigated the effects of self-directed respiratory muscle training in this context by assessing pulmonary function relative to spinal cord injury characteristics. A total of 104 spinal cord injury patients (tetraplegia/paraplegia; 65/39, acute/subacute/chronic; 14/42/48) were admitted for short-term (4–8 weeks) in-patient clinical rehabilitation. Initial evaluation revealed a compromised pulmonary function with a percentage of predicted value of 62.0 and 57.5 in forced vital capacity in supine and forced vital capacity in sitting positions, respectively. Tetraplegic patients had more compromised pulmonary function compared with paraplegic patients. At follow-up evaluation, the percentage of predicted value of forced vital capacity in supine and sitting position improved overall on average by 11.7% and 12.7%, respectively. The peak cough flow improved by 22.7%. All assessed pulmonary function parameters improved significantly in all subgroups, with the greatest improvements found in patients with tetraplegia and subacute spinal cord injury. Therefore, short-term self-directed respiratory muscle training should be incorporated into all spinal cord injury rehabilitation regimens, especially for patients with tetraplegia and subacute spinal cord injury, as well as those with chronic spinal cord injury.

## Introduction

Cervical and upper thoracic spinal cord injury (SCI) is associated with variable degrees of pulmonary dysfunction resulting from impairments in respiratory muscle performance, such as the diaphragm and intercostal, accessory respiratory, and abdominal muscles. Debilitation of these muscles renders deep breathing difficult for affected individuals, causing atelectasis and reduced compliance of the lung and chest wall. Additionally, compromised expiratory muscle function prevents the effective clearance of airway secretions. These limitations in pulmonary function (PF) result in respiratory complications, mucus retention, pneumonia, and eventually, respiratory failure^1,2^. Although the PF gradually improves with neurological recovery, respiratory complications are the primary cause of morbidity and mortality among patients with SCI^3,4^.

In order to reduce respiratory complications in SCI patients, early initiation of respiratory care with respiratory muscle training (RMT) is essential^5^. Previous studies investigating RMT reported significant improvements in the strength and endurance of respiratory muscles, ameliorating respiratory complications in response to this training^6–9^. However, the significant implications of regular respiratory care are often ignored in most SCI cases, particularly in patients not requiring a ventilator. Moreover, besides the fact that most previous studies were conducted with small sample sizes (<50 patients), there is lack of evidence on how SCI characteristics may affect the degree of improvement in respiratory function.

Therefore, this study aimed to investigate the effect of self-directed RMT by SCI patients during short-term in-patient rehabilitation. Importantly, we examined potential differences in the degree of improvement in PF depending on the characteristics of the SCI including injury level and severity, as well as disease duration.

## Methods

### Participants

This retrospective study involved 178 SCI patients who were admitted to the tertiary university hospital for a short-term (4–8 weeks) rehabilitation treatment between December 2016 and November 2017.

Only those patients with available records of initial and follow-up pulmonary evaluation, including forced vital capacity in supine position (FVCsup) and sitting position (FVCsit), and peak cough flow (PCF), were included. The exclusion criteria were as follows: (1) tracheostomy or gastrostomy status on admission; (2) underlying neurological conditions other than SCI such as stroke, traumatic brain injury, and degenerative brain diseases; (3) medical history affecting PF such as chronic obstructive pulmonary disease, bronchial asthma, and combined chest wall injury during SCI; and (4) cognitive impairment, with a score of <25 in the Korean version of the Mini-Mental State Examination.

Sixty-eight patients were excluded owing to lack of initial or follow-up PF test results. In addition, 3 patients with tracheostomy or gastrostomy, 1 patient who had undergone previous surgery for the treatment of lung cancer, and 2 patients with rib fractures were excluded. Finally, 104 patients with SCI were included. Medical chart review was used to obtain information on sex; age; past medical history including the onset, cause, level of injury, and injury severity; and PF records of each patient.

Participants were classified into different groups as per the American Spinal Injury Association Impairment Scale based on injury level as tetraplegic or paraplegic, by injury severity as complete or incomplete, and by disease duration as acute (<2 weeks), subacute (2–8 weeks), or chronic (>8 weeks).

This study was conducted in accordance with the recommendations of the institutional review board of Yonsei University Health System, which also approved the protocol used in this study. Given the retrospective study design, the need for informed consent was waived (ethical approval number: 4–2018–0990).

### Pulmonary function evaluation

Evaluation of PF was performed weekly during hospitalisation by 2 physicians within the rehabilitation ward. The FVC and PCF were used as parameters for PF evaluation, with both FVCsit and FVCsup measured using a hand-held spirometer (Micro Medical Ltd., Rochester, Kent, UK). At least 3 measurements were obtained per patient with 2-minute intervals between individual assessments, and the maximum value was selected. The FVC is expressed as the percentage of the predicted normal value (%pre)^10,11^.

Patients underwent PCF measurement using a peak-flow meter (Philips Respironics, Guildford, UK) in the sitting position. Unassisted PCF was assessed by having the patients cough as forcefully as possible through the peak-flow meter, whereas assisted PCF was measured with the tester’s thrusting into the patient’s abdomen when coughing. With the indicator positioned at the bottom of the device’s meter scale, the patients were asked to produce a single cough. The maximum observed flow in at least 3 attempts was recorded.

All PF parameters were assessed and analysed before and after the short-term rehabilitation therapy, with respective changes expressed as ΔFVCsup, ΔFVCsit, and ΔPCF.

### Respiratory muscle training and care

Respiratory muscle training and care consisted of glossopharyngeal breathing exercise, inspiratory muscle strengthening using incentive spirometry, and air stacking exercises with a resuscitation bag. On admission, a physiatrist and a physical therapist in charge of pulmonary rehabilitation trained all patients accordingly. The physical therapist evaluated all patients’ exercises on a weekly basis and corrected inadequacies.

Inspiratory muscle strengthening was performed using incentive spirometry (Coach 2 device^®^, MediMark Europe, Grenoble, France) in an upright sitting position. The patients were instructed to breathe through the mouthpiece slowly and as deeply as possible until the yellow piston indicator had reached the outlined area, to hold the breath for at least 5 seconds, and exhale slowly subsequently. A total of 2 sets of 20 repetitions each were conducted by all patients per day, more than 5 days a week. After each set, a 1-minute rest was allowed. In case of fatigue or dizziness during the RMT, a short rest was allowed^12,13^.

Glossopharyngeal breathing exercise were performed 10 times a day, more than 5 days a week. Glossopharyngeal breathing exercise is a complex process involving the mouth, cheeks, lips, tongue, soft palate, larynx, and pharynx to piston boluses of air into the lungs. To increase the lung volume above the total lung capacity, the patients inhale, hold the air, and subsequently swallow cycles of as many gulps of air as possible, prior to relaxing the larynx by passive expulsion of the air^14^.

The air stacking exercise was performed as follows: after the inhalation of the maximum amount of air, the caregiver infused additional air into the subject’s lungs about 2–3 times using a resuscitation bag connected to an oro-nasal mask. The air was held for several seconds before the patient was allowed to exhale passively or to cough. In case patients lacked sufficient PCF for eliminating secretion, an assisted coughing method by air stacking and abdominal thrust was taught^15^. This exercise was conducted 3–5 times per session, twice a day, more than 5 days a week.

### Statistical analyses

Statistical analyses were performed using a standard statistical software (SPSS version 21.1 for Windows, SPSS, Inc., Chicago, IL, USA). Values are reported as mean ± standard deviation, and P values of < 0.05 were considered to be statistically significant. Absolute values of PF prior to and following short-term rehabilitation, as well as potential changes (Δ) in PF between the two time points, were analysed in correlation with patient characteristics using the Wilcoxon and paired t test. For comparisons related to disease duration, one-way ANOVA with Bonferroni post-hoc test was used. Pearson and Spearman correlation coefficients were calculated to determine potential improvements in PF.

## Results

### General characteristics and absolute values of pulmonary function

Detailed characteristics of the study population are presented in Table 1. The average age was 48.7 ± 17.5 years, with most participants being male (75%). The majority of patients were tetraplegic (62.5%), while 90.4% had a traumatic background and 79.8% of the injuries were incomplete.

**Table 1:** Demographics and clinical characteristics.

Characteristics (%)	N = 104
Age (years, range)	48.7 ± 17.5 (15–84)
Male/female	78/26 (75.0/25.0)
**Aetiology of spinal cord injury**	
Trauma	94 (90.4)
Vascular	4 (3.8)
Tumour	3 (2.9)
Infection	3 (2.9)
Tetraplegia/paraplegia	65/39 (62.5/37.5)
AIS A/B/C/D	21/7/30/46 (20.2/6.7/28.8/44.2)
Complete/Incomplete	21/83 (20.2/79.8)
Disease duration (day, range)	97.4 ± 139.2 (7–993)
**Acute/subacute/chronic**	14/42/48 (13.5/40.4/46.2)
Acute (tetraplegia/paraplegia)	9/5 (64.3/35.7)
Subacute (tetraplegia/paraplegia)	30/15 (66.7/33.3)
Chronic (tetraplegia/paraplegia)	26/19 (57.8/42.2)
Height (cm, range)	168.3 ± 8.8 (129–186)
Body weight (kg, range)	64.6 ± 11.4 (24–95)
Body mass index (kg/m^2^, range)	22.7 ± 3.2 (13.9–33.4)

Values are mean ± standard deviation. (% or range).

AIS, ASIA Impairment Scale.

**Figure 1:**
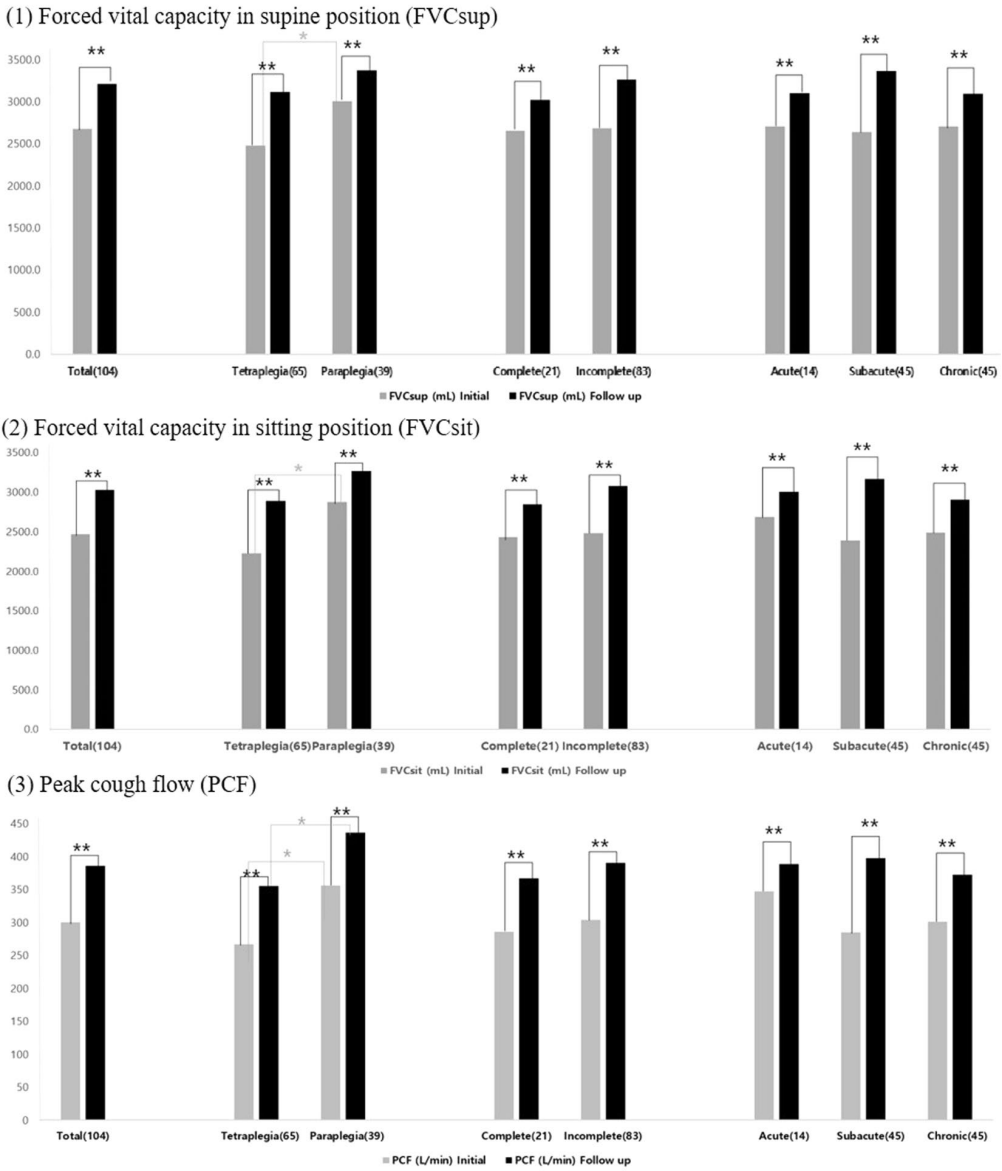
Inter- and intra-group comparisons of forced vital capacity (FVC) and peak cough flow (PCF) before and after short-term rehabilitation according to the level of injury, injury severity, and disease duration. *P < 0.05, **P < 0.01.

Initial evaluation revealed a compromised PF with a percentage of predicted value (%pre) of 62.0 and 57.5%pre in FVCsup and FVCsit positions, respectively (Tables 2, 3). All assessed parameters including FVCsup, FVCsit, and PCF were more severely affected in the tetraplegic group compared to the paraplegic group (P < 0.01, Tables 2–4, Fig. 1).

**Table 2:** Change in forced vital capacity in the supine position before and after short-term rehabilitation according to the level of injury, injury severity, and disease duration.

(N)	FVCsup (mL)					pre%FVCsup (%)		
	Initial	Follow up	Δ (%)	*p*		Initial	Follow up	Δ
				Intra group	Inter group			
Total (104)	2673.0 ± 879.8	3209.0 ± 883.0	536.1 ± 491.0 (24.3)	<0.001**		62.0 ± 18.9	74.3 ± 17.8	12.3 ± 10.6
**Level of injury**								
Tetraplegia (65)	2474.9 ± 758.7	3112.3 ± 860.5	637.4 ± ± 518.80 (29.3)	<0.001**	0.003^††^	57.5 ± 16.9	72.1 ± 18.2	14.6 ± 10.9
Paraplegia (39)	3003.1 ± 974.5	3370.3 ± ± 907.6	367.2 ± 391.4 (16.0)	<0.001**		69.5 ± 19.8	78.0 ± 16.8	8.5 ± 9.1
**Injury severity**								
Complete (21)	2649.1 ± 1024.0	3018.1 ± 1037.0	369.1 ± 369.3 (18.2)	<0.001**	0.039*	56.2 ± 19.5	64.3 ± 19.7	8.1 ± 7.9
Incomplete (83)	2679.0 ± 846.4	3257.4 ± 839.9	578.3 ± 510.4 (25.9)	<0.001**		63.5 ± 18.5	76.9 ± 16.5	13.4 ± 11.0
**Disease duration**								
Acute (14)	2705.0 ± 964.1	3095.7 ± 1081.5	390.7 ± 433.2 (15.7)	<0.001**	0.002^††^	65.7 ± 21.2	73.4 ± 22.9	9.6 ± 9.8
Subacute (45)	2634.9 ± 944.8	3365.3 ± 877.0	730.4 ± 485.8 (33.8)	<0.001**		53.7 ± 20.4	71.0 ± 17.6	16.3 ± 10.2
Chronic (45)	2701.1 ± 801.3	3088.0 ± 816.3	386.9 ± 454.4 (17.6)	<0.001**		58.8 ± 19.4	68.4 ± 19.7	9.1 ± 10.1

Values are mean ± standard deviation.

**p < 0.01 by paired t test, ^††^*p* < 0.01 by Wilcoxon test.

FVC, forced vital capacity; FVCsup, FVC in supine position; pre%, percentage of the predicted normal value; Δ, change.

**Table 3:** Change in forced vital capacity in the sitting position before and after short-term rehabilitation according to the level of injury, injury severity, and disease duration.

(N)	FVCsup (mL)					pre%FVCsup (%)		
	Initial	Follow up	Δ (%)	*p*		Initial	Follow up	Δ
				Intra group	Inter group			
Total (104)	2468.9 ± 922.2	3028.9 ± 930.8	560.0 ± 507.5 (28.9)	<0.001**		57.5 ± 20.3	70.2 ± 19.2	12.3 ± 10.6
**Level of injury**								
Tetraplegia (65)	2226.5 ± 784.1	2888.5 ± 887.1	662.0 ± 537.5 (36.0)	<0.001**	0.004††	52.1 ± 18.2	67.1 ± 19.	15.0 ± 11.3
Paraplegia (39)	2872.8 ± 1000.9	3262.8 ± 965.8	390.0 ± 405.1 (17.2)	<0.001**		66.5 ± 20.2	75.4 ± 18.1	8.9 ± 9.4
**Injury severity**								
Complete (21)	2429.5 ± 1003.1	2844.8 ± 1021.2	415.2 ± 359.5 (20.5)	<0.001**	0.072	51.6 ± 19.4	60.5 ± 19.6	8.9 ± 7.4
Incomplete (83)	2478.8 ± 906.8	3075.4 ± 907.2	596.6 ± 534.1 (31.1)	<0.001**		59.0 ± 20.3	72.6 ± 18.4	13.7 ± 11.5
**Disease duration**								
Acute (14)	2687.1 ± 1017.8	3005.7 ± 1139.4	318.6 ± 434.8 (12.9)	<0.001**	0.002††	65.7 ± 21.2	73.4 ± 22.9	7.7 ± 9.1
Subacute (45)	2385.1 ± 999.1	3165.8 ± 940.1	780.7 ± 503.3 (42.3)	<0.001**		53.7 ± 20.4	71.0 ± 17.6	17.4 ± 10.4
Chronic (45)	2484.7 ± 814.8	2899.1 ± 851.0	414.4 ± 451.5 (20.6)	<0.001**		58.8 ± 19.4	68.4 ± 19.7	9.6 ± 10.4

Values are mean ± standard deviation.

**p < 0.01 by paired t test, ^††^*p* < 0.01 by Wilcoxon test.

FVC, forced vital capacity; FVCsit, FVC in sitting position; pre%, percentage of the predicted normal value; Δ, change.

**Table 4:** Change in peak cough flow before and after short-term rehabilitation according to the level of injury, injury severity, and disease duration.

(N)	FVCsup (mL)				
	Initial	Follow up	Δ (%)	*p*	
				Intra group	Inter group
Total (104)	300.2 ± 145.4	385.6 ± 145.7	85.4 ± 82.9 (63.4)	<0.001**	
**Level of injury**					
Tetraplegia (65)	266.9 ± 136.8	355.2 ± 137.5	88.3 ± 78.9 (82.1)	<0.001**	0.003††
Paraplegia (39)	355.8 ± 144.0	436.2 ± 146.7	80.4 ± 90.0 (32.1)	<0.001**	
**Injury severity**					
Complete (21)	285.7 ± 140.0	366.7 ± 150.2	81.0 ± 69.0 (35.7)	<0.001**	0.653
Incomplete (83)	303.9 ± 147.4	390.4 ± 145.1	86.5 ± 86.4 (70.4)	<0.001**	
**Disease duration**					
Acute (14)	347.1 ± 136.1	388.6 ± 136.0	41.4 ± 53.9 (14.9)	<0.001**	0.004††
Subacute (45)	284.6 ± 150.0	398.0 ± 157.8	113.4 ± 94.8 (104.8)	<0.001**	
Chronic (45)	301.3 ± 143.4	372.3 ± 137.9	70.9 ± 67.9 (37.0)	<0.001**	

Values are mean ± standard deviation.

**p < 0.01 by paired t test, ^††^*p* < 0.01 by Wilcoxon test.

PCF, peak cough flow; %ΔPCF, percent change of peak cough flow; L/min, litre per minute.

The absolute value of FVCsup was significantly higher compared with that of FVCsit at the initial and final assessment of the short-term rehabilitation therapy in all subgroups, except for the acute group (Table 5).

**Table 5:** Comparison between forced vital capacity in the supine position and forced vital capacity in the sitting position before and after short-term rehabilitation according to the level of injury, injury severity, and disease duration.

(N)	Initial			Follow up		
	FVCsup (mL)	FVCsit (mL)	*p*	FVCsup (mL)	FVCsit (mL)	*p*
Total (104)	2673.0 ± 879.8	2468.9 ± 922.2	<0.001**	3209.0 ± 883.0	3028.9 ± 930.8	<0.001**
**Level of injury**						
Tetraplegia (65)	2474.9 ± 758.7	2226.5 ± 784.1	<0.001**	3112.3 ± 860.5	2888.5 ± 887.1	0.001**
Paraplegia (39)	3003.1 ± 974.5	2872.8 ± 1000.9	0.001**	3370.3 ± 907.6	3262.8 ± 965.8	0.002**
**Injury severity**						
Complete (21)	2649.1 ± 1024.0	2429.5 ± 1003.1	<0.001**	3018.1 ± 1037.0	2844.8 ± 1021.2	<0.001**
Incomplete (83)	2679.0 ± 846.4	2478.8 ± 906.8	0.011*	3257.4 ± 839.9	3075.4 ± 907.2	<0.001**
**Disease duration**						
Acute (14)	2705.0 ± 964.1	2687.1 ± 1017.8	0.747	3095.7 ± 1081.5	3005.7 ± 1139.4	0.119
Subacute (45)	2634.9 ± 944.8	2385.1 ± 999.1	<0.001**	3365.3 ± 877.0	3165.8 ± 940.1	<0.001**
Chronic (45)	2701.1 ± 801.3	2484.7 ± 814.8	<0.001**	3088.0 ± 816.3	2899.1 ± 851.0	<0.001**

Values are mean ± standard deviation.

*p < 0.05, **p < 0.01 by paired t test.

FVC, forced vital capacity; FVCsup, FVC in supine position; FVCsit, FVC in sitting position.

### Changes in pulmonary function after short-term rehabilitative treatment

Following the short-term rehabilitation treatment with self-directed RMT, the absolute values of FVCsup, FVCsit, and PCF had significantly improved in all subgroups regardless of the injury level and severity, as well as disease duration (Tables 2–4, Fig. 1).

The mean values (mL) of ΔFVCsup were 637.4 ± 518.8 (25.8%) in tetraplegic and 367.2 ± 391.4 (12.2%) in paraplegic patients; 369.1 ± 369.3 (18.2%) in complete and 578.3 ± 510.4 (25.9%) in incomplete groups; and 390.7 ± 433.2 (15.7%) in acute, 730.4 ± 485.8 (33.8%) in subacute, and 386.9 ± 452.4 (17.6%) in chronic groups.

The mean values (mL) of ΔFVCsit were 662.0 ± 537.5 (29.7%) in tetraplegic and 390.0 ± 405.1 (13.6%) in paraplegic patients; 359.5 ± 534.1 (20.5%) in complete and 596.6 ± 534.1 (31.1%) in incomplete groups; and 318.6 ± 434.8 (12.9%) in acute, 780.7 ± 503.3 (42.3%) in subacute, and 414.4 ± 421.5 (20.6%) in chronic groups.

The mean values (L/min) of ΔPCF were 88.3 ± 78.9 (82.1%) in tetraplegic and 80.4 ± 90.0 (32.1%) in paraplegic patients; 81.0 ± 69.0 (37.5%) in complete and 86.5 ± 86.4 (70.4%) in incomplete groups; and 41.4 ± 53.9 (14.9%) in acute, 113.4 ± 94.8 (104.8%) in subacute, and 70.9 ± 67.9 (37.8%) in chronic groups.

**Figure 2:**
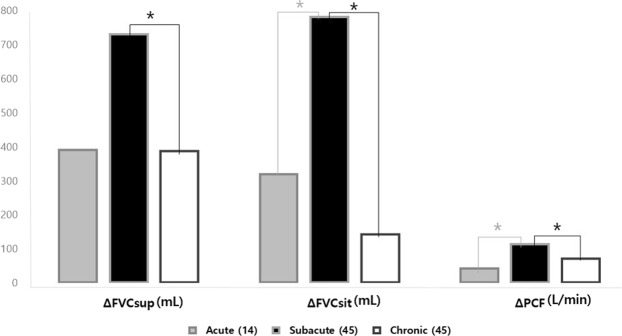
Comparisons of changes in each parameters of pulmonary function before and after short-term rehabilitation according to the disease duration. *P < 0.05, ANOVA with Bonferroni post-hoc test. FVC, forced vital capacity; FVCsup, FVC in supine position; FVCsit, FVC in sitting position; Δ, change.

We found significant inter-group differences in ΔFVCsup in all subgroups (P < 0.05, Table 2, Fig. 2). In addition, ΔFVCsit and ΔPCF showed significant inter-group differences with regard to the level of injury and disease duration (P < 0.01, Tables 3, 4, Fig. 2). In other words, the subacute group showed the highest improvement in ΔFVCsit and ΔPCF, compared with the acute and chronic groups (P < 0.05, Fig. 2). The subacute group was also observed to have a greater ΔFVCsup compared with the chronic group (P = 0.002) and a higher tendency compared with the acute group in this context (P = 0.056, Fig. 2).

The percentages of ΔFVCsup, ΔFVCsit, and ΔPCF, which were found to have a significantly positive correlation with each other, exhibited a negative correlation with the baseline values of FVCsup, FVCsit, and PCF, respectively (P < 0.05, Table 6).

**Table 6:** Correlation between the initial pulmonary function and the percent improvement of pulmonary function.

	Initial FVCsup	Initial FVCsit	Initial PCF	%ΔFVCsup	%Δ FVCsit	%ΔPCF
%ΔFVCsup	−0.531**	−0.510**	−0.428**	1	0.795**	0.499**
%ΔFVCsit	−0.386**	−0.513**	−0.414**	0.795**	1	0.550**
%ΔPCF	−0.209*	−0.248*	−0.328**	0.499**	0.550**	1

Values are Pearson correlation coefficient r.

*P < 0.05, **P < 0.01.

FVC, forced vital capacity; FVCsup, FVC in supine position; FVCsit, FVC in sitting position; Δ, change.

## Discussion

While the present study confirmed the impaired PF in SCI patients, it also revealed that short-term rehabilitative treatment with self-directed respiratory care had the capacity to induce significant improvements in the respiratory function of these patients. This beneficial effect was consistently observed among all subgroups with significant inter-group differences, the only exception being the injury severity group.

Immediately following SCI, PF is compromised and may recover slowly over several months to a year^16–19^. However, PF may also deteriorate at any point of time in SCI patients, and similar to the increasing susceptibility of aging individuals, the decline in PF in SCI patients is more rapid compared with able-bodied individuals^20,21^. This observation may be mainly due to the lack of physical activity in the majority of these patients^22,23^. Moreover, reduced PF in SCI patients is associated with an increased risk of respiratory illness as well as higher mortality rates, and FVC can be a predictor of respiratory infection in the first year post discharge^24,25^. Therefore, appropriate respiratory care for optimising PF may be crucial to improve physical fitness and survival in these patients. However, conventional rehabilitation therapy through physical and occupational therapy often neglects regular respiratory care.

The main goals of the respiratory care program are to maintain lung compliance and increase chest expansion by air stacking^15^ and glossopharyngeal breathing exercise^2^, to strengthen the inspiratory muscles for facilitation of breathing with lower energy expenditure^9^, and to facilitate the prompt elimination of pulmonary secretions^26^. The ability to breathe deeply and to cough to eliminate secretions depend on the neurological injury level. Therefore, the rehabilitative respiratory care applied in this study comprised active strengthening exercises, passive air stacking, as well as assisted coughing by abdominal thrust and glossopharyngeal breathing exercise. The physiatrist and physical therapist trained the patients and caregivers in self-directed respiratory care. SCI patients as well as caregivers participated in these exercises actively for 20–30 minutes per day, leading to a significant improvement in the PF of the patients after only 4–8 weeks of rehabilitation. During this time, the physiatrist reassessed the patients’ PF once a week to ensure the adequacy and accuracy of respiratory care performed by the patients and caregivers.

Previous studies have reported that patients with cervical SCI have a better FVCsup as compared with FVCsit^27^, owing to the fact that the diaphragm increases its inspiratory excursion in a supine position, whereas this process is negatively affected by gravity due to abdominal weakness in a sitting position^2^. The pattern of FVCsup and FVCsit in the participants of the present study, except for the acute group, was consistent with previous studies, confirming the reliability and reproducibility of our data (Table 5).

All assessed PF parameters improved in all subgroups following self-directed RMT. The predicted FVC improved overall on average by 11.7% (from 62.6% to 74.3%) in the supine position (Table 2) and by 12.7% (from 57.5% to 70.2%) in the sitting position (Table 3), which is consistent with a previously published average of 12.4% (from 71.7% to 84.1%) in the predicted FVC during in-patient rehabilitation4. A Recent study^28^ utilizing repeated maximal expiration and inspiration training and abdominal drawing-in maneuver improved PF, especially FVCsit by 20.0%. On the other hand, in our study, overall improvement of FVCsit after RMT was estimated as high as 28.9%, which varied from 12.9% to 42.3% depending on the sub-groups. These results suggest that various RMT components may contribute to produce various degrees of therapeutic effects on PF, requiring a lot of follow-up studies to decide the most effective method.

The significance of our study lies in showing the varying effects of RMT on PF depending on the characteristics of SCI patients. Moreover, despite the initially low absolute values, the tetraplegic group revealed significant improvements in FVCsup, FVCsit, and PCF compared with the paraplegic group (Tables 2–4). The percent improvement observed in these 3 parameters showed a significant negative correlation with their respective baseline values. These results indicate that RMT was more beneficial in patients with poor PF and suggest that the active implementation of RMT has a strong clinical value, especially in this group of patients.

Significant improvements in FVCsup, FVCsit, and PCF were also observed in the subacute group as compared with the acute and chronic groups (Tables 2–4). These observations may be attributed to the greater effect of training and the highest degree of natural neurological recovery in the subacute stage of SCI. It is also noteworthy that the average %ΔFVCsup, %ΔFVCsit, and %ΔPCF measured in the chronic group were as high as 17.6%, 20.6%, and 37.8%, respectively (Tables 2–4). Our observation of a significant amelioration in PF in the chronic group, especially in PCF, may have resulted from a previous lack of respiratory care or other rehabilitative interventions among these patients. Consequently, although RMT demonstrates comparatively more favourable outcomes in the subacute phase of SCI, chronic SCI patients may nevertheless still substantially benefit from and should therefore be considered for RMT, including self-directed RMT.

Inter-group analysis based on the injury level revealed significantly more compromised baseline PF in the tetraplegic group compared to the paraplegic group (Fig. 1). After short-term rehabilitation, both groups showed significant improvement in all parameters of PF and no more inter-group differences in FVCsup and FVCsit could be detected (Fig. 1). However, on follow-up, the tetraplegic group still showed significantly lower PCF results compared with the paraplegic patients (Fig. 1). This may be explained by the fact that FVC primarily involves the inspiratory muscle, whereas PCF involves both inspiratory and expiratory muscles. As tetraplegic patients tend to have weaker inspiratory and expiratory muscles, more emphasis should be placed on improving PCF during training in these patients, also to ensure proper elimination of secretions.

Our results imply that education and training in self-directed RMT can be an effective means to enhance respiratory function. To the best of our knowledge, our study compared, for the first time, the effect of RMT on the improvement in PF in patients with tetraplegia and paraplegia caused by SCI, taking into account SCI characteristics. Thus, our findings provide basic scientific data for PF in SCI patients relative to various disease-specific features, and could therefore serve as the indicators of RMT. Moreover, this self-directed RMT program may be used as a standard model to maintain or improve the PF not only for in-patients, but also for out-patients who have difficulties visiting a hospital.

This study has several limitations. Firstly, it does not assess the effects of improved PF on the level of physical function, which should be addressed in future studies. Secondly, due to the absence of a control group, this study could not distinguish the improvement in PF contributed by RMT from that conferred by neurological recovery. Finally, the history of smoking, presence of tracheostomy, or use of mechanical ventilator was not considered in this study. Therefore, further studies with larger numbers of SCI patients taking into account various injury characteristics (level, severity, and duration) and clinical information (smoking, tracheostomy, and use of ventilator) will be needed to identify the factors contributing to PF improvement and, ultimately, to a favourable rehabilitative outcome.

In conclusion, short-term rehabilitation treatment with self-directed RMT resulted in the improvement of PF in all SCI groups. Based on these findings, the need for RMT to be incorporated into all SCI rehabilitation programs must be emphasised. In particular, tetraplegic and subacute SCI patients have had the greatest benefit from RMT. Owing to the observed positive effects of RMT in chronic SCI patients, its use must be actively considered even in the later stages in chronic SCI patients.

## Data Availability

Data are available by requesting the corresponding author, Sang Hee Im.
